# Deep learning with microfluidics for on-chip droplet generation, control, and analysis

**DOI:** 10.3389/fbioe.2023.1208648

**Published:** 2023-06-07

**Authors:** Hao Sun, Wantao Xie, Jin Mo, Yi Huang, Hui Dong

**Affiliations:** ^1^ School of Mechanical Engineering and Automation, Fuzhou University, Fuzhou, China; ^2^ Fujian Provincial Collaborative Innovation Center of High-End Equipment Manufacturing, Fuzhou, China; ^3^ Centre for Experimental Research in Clinical Medicine, Fujian Provincial Hospital, Fuzhou, China

**Keywords:** droplet microfluidics, deep learning, artificial intelligence, intelligent microfluidics, on-chip analysis

## Abstract

Droplet microfluidics has gained widespread attention in recent years due to its advantages of high throughput, high integration, high sensitivity and low power consumption in droplet-based micro-reaction. Meanwhile, with the rapid development of computer technology over the past decade, deep learning architectures have been able to process vast amounts of data from various research fields. Nowadays, interdisciplinarity plays an increasingly important role in modern research, and deep learning has contributed greatly to the advancement of many professions. Consequently, intelligent microfluidics has emerged as the times require, and possesses broad prospects in the development of automated and intelligent devices for integrating the merits of microfluidic technology and artificial intelligence. In this article, we provide a general review of the evolution of intelligent microfluidics and some applications related to deep learning, mainly in droplet generation, control, and analysis. We also present the challenges and emerging opportunities in this field.

## 1 Introduction

### 1.1 Droplet microfluidic technology

Microfluidics is a system capable of processing or manipulating small quantities of fluids (ranging from 10^−9^ to 10^−18^ L) in a microscale structure (Wang et al., 2020). The system can precisely control liquids at the micrometer or nanometer level, and the fluids produce totally different effects from macro-fluids, such as large laminar flow and high heat transfer efficiency (Yang et al., 2020). Due to these features, microfluidic technology has rapidly evolved in recent decades, bringing a new perspective to many traditional disciplines and showing great potential in fields such as biology, chemistry, medicine, energy, and materials (Sun et al., 2015; Chowdhury et al., 2019; Zhang et al., 2019; Jia et al., 2020; Li et al., 2021a; Zheng et al., 2021). Additionally, microfluidic systems offer the advantages of high throughput, high sensitivity and low power consumption (Sun et al., 2022). Moreover, they are capable of generating large amounts of data, including size, shape, composition, and other parameters (Isozaki et al., 2020). Therefore, microfluidics has become an interdisciplinary discipline involving engineering, physics and micro-processing.

Droplet microfluidics is a subfield of microfluidics that focuses on the manipulation and control of droplets in micro-scale channels. It involves the precise handling of droplets to perform various chemical and biological assays, as well as the development of methods to generate, merge, and split droplets (Postek and Garstecki, 2022). The basic principles of droplet microfluidics include the use of microfluidic channels and small quantities of fluids to create, manipulate, and analyze droplets (Elvira et al., 2022). Typically, droplets are produced by fragmenting a continual fluid stream into small, uniform droplets employing a diverse array of techniques. The function of generating and operating microdroplets can be achieved by exploiting the different physical and chemical characteristics of multiphase fluids as they flow through microchannels and microstructures in microfluidic chips (Sun et al., 2020). Each microdroplet can be considered an independent reaction unit, as the droplets are separated and do not merge with each other, thereby avoiding cross-contamination (Yu et al., 2022).

Due to its outstanding advantages, droplet microfluidics is widely used in various fields, particularly in the fields of chemical analysis and life sciences. It enables single-cell manipulation and highly controlled dynamic monitoring (Chen et al., 2019). Novel droplet-based molecular biology techniques have been developed to detect cellular matter, including DNA, RNA, proteins and other metabolites (Contreras-Naranjo et al., 2017). Droplet microfluidic technology has also revolutionized many standard molecular biology techniques, providing new technology platforms for polymerase chain reaction (PCR), reverse transcription PCR (RT-PCR), enzyme-linked immunosorbent assays (ELISA), and more (Moon et al., 2018; Li et al., 2019). Furthermore, it has a wide range of applications in high-throughput drug screening, microcapsule synthesis, and single-molecule analysis (Kobayashi et al., 2019). In addition, droplet microfluidics is useful for environmental analysis and may potentially produce functional materials with unprecedented characteristics that are difficult to obtain using traditional synthesis methods (Hou et al., 2017; Kung et al., 2019).

### 1.2 Microfluidics integrated with machine learning and deep learning

Machine learning is a class of artificial intelligence (AI)-based methods that direct computers to learn rules from data and then use the experience to improve their performance without explicit programming. It was first proposed as a research area at the Dartmouth Conference in 1956 (Haenlein and Kaplan, 2019). However, research interests in AI were limited at that time due to the low capability of computers in information storage and processing. Then, data-driven machine learning came back to life and gradually became the major application of AI in the late 20th century, making great contributions to the development of computer science (Fradkov, 2020). At the turn of the 21st century, with the improvement of computing power as well as the abundance of available data, academic research related to machine learning became unprecedentedly active and the range of applications via various learning methods constantly expanded (Molnar et al., 2020). Although traditional machine learning has long provided strong assistance for data processing tasks, the emergence of deep learning methods greatly enhances computers’ ability in dealing with huge and complicated datesets (Paullada et al., 2021).

Deep learning is a subset of machine learning proposed in the 2010s (Wang and Raj, 2017). It was introduced in order to help people get closer to artificial intelligence and has received enormous attention in a wide range of applications due to its powerful learning ability (Alzubaidi et al., 2021). Deep learning realizes the feature extraction of input data from low-level to high-level by establishing and simulating the neural structure of the human brain for information processing. This allows the machine to understand and learn from the data, and then obtain information (Jogin et al., 2018). Since the Alexnet (Krizhevsky et al., 2017) achieved amazing results in the ImageNet competition, a large amount of research has been done to improve the performance of different networks such as convolutional neural networks (CNN) and recurrent neural network (RNN) (Yu et al., 2019; Dhillon and Verma, 2020). With the rapid development in the past decade, deep learning architectures can now handle structured data obtained from various research fields. It has recently made great achievements in the analysis of data in different domains, including images (Wu et al., 2020; Minaee et al., 2021a), sound (Raza et al., 2019; Grumiaux et al., 2021), natural language (Otter et al., 2020; Feghali et al., 2022) and text documents (Minaee et al., 2021b; Long et al., 2021). On the other hand, these achievements have also been contributed to by the increasing computing ability of GPUs and the popularity of open-source frameworks such as TensorFlow (Abadi et al., 2016) and PyTorch (Paszke et al., 2019).

**FIGURE 1:**
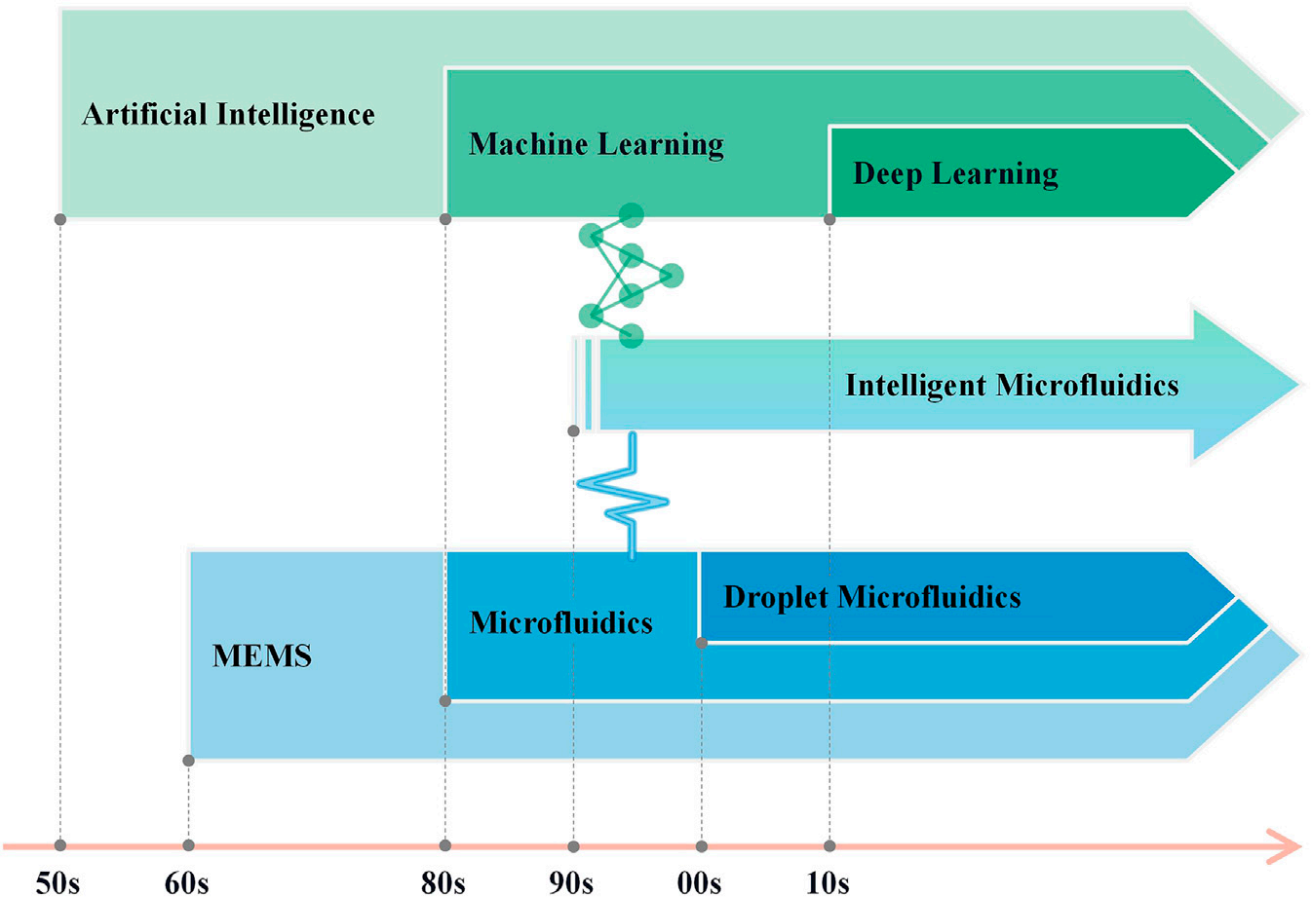
Timeline of the development of intelligent microfluidics.

The high throughput of microfluidics enables the generation of massive and detailed data. Compared to traditional methods of data analysis that rely more on human intervention, deep learning utilizes large amounts of data for feature extraction, requiring less manual intervention to improve the performance of computer-aided tasks such as classification and prediction of data from microfluidic systems (Sun et al., 2023a). Microfluidics and deep learning-based data analysis are combined to provide a great deal of new ideas for related research. Intelligent microfluidics has shown its ability to solve problems that are hard or next to impossible for traditional methods, such as label-free biomedical detection (Kobayashi et al., 2017) and exploration of optimum conditions for specific reactions (Zhou et al., 2017). In addition, microfluidic systems and the introduced AI models can provide feedback to each other, which is conducive to the optimization of both sides and significant for achieving the automation and intelligence of microfluidic systems (de Almeida et al., 2019; Uddin et al., 2019). Therefore, there will be very broad space for the development and application of microfluidics integrated with AI in the future. Figure 1 shows the evolution of intelligent microfluidics. In this section, we provided an overview of the evolution of intelligent microfluidics. Subsequently, we elaborated on the utilization of intelligent microfluidics, focusing on droplet generation, control, and analysis. Lastly, we outlined the hurdles and fresh prospects confronting this field, with the aspiration of inspiring novel research notions for researchers.

## 2 Applications in microfluidics with deep learning

### 2.1 Droplet generation and chip design

The advent of droplet-based microfluidic devices has paved the way for the application of lab-on-a-chip (LoC) concept. As an essential part of LoC experiments, the generation of microdroplets forms the basis for the extensive use of droplet microfluidic technology (Hettiarachchi et al., 2021). However, the droplets differ significantly in characteristics such as droplet shape and size, depending on the structure of the microchannels (Lei et al., 2021). In single-cell analysis, precise cell encapsulation and minimal cross-contamination of droplets are necessary to ensure the accurate identification of individual cells (Chen et al., 2021). Similarly, in digital PCR, specific droplet features such as uniform size and high encapsulation efficiency are essential for accurate and reliable measurements (Park et al., 2021). Since specific droplet features are required for research and applications in different fields integrated with microfluidics, the design of chips has become a time-consuming and laborious matter. With the progress in artificial intelligence, such works can now be accomplished more efficiently with the aid of computers (Lashkaripour et al., 2019).

To predict the dimensionless length of water-infused droplets in microfluidic systems, Mahdi and Daoud (2017) employed an artificial neural network (ANN), considering factors like flow rate, viscosity, and microchannel diameter as inputs. The network was trained using the average length of droplets measured from 150 images, and showed a high level of accuracy when compared to the experimental data. Lashkaripour et al. (2018) developed ANFIS (adaptive neural-fuzzy inference system) to predict droplet size in a microfluidic flow-focusing junction based on geometry, flow, and fluid properties. Six parameters including orifice width and surface tension were considered during the training of the model, and a significant accuracy of 96% was achieved. Similarly, Mottaghi et al. (2020) conducted a study exploring the impact of four dimensionless parameters on droplet size, namely, Ca, Re, flow rate ratio, and viscosity ratio.

**FIGURE 2:**
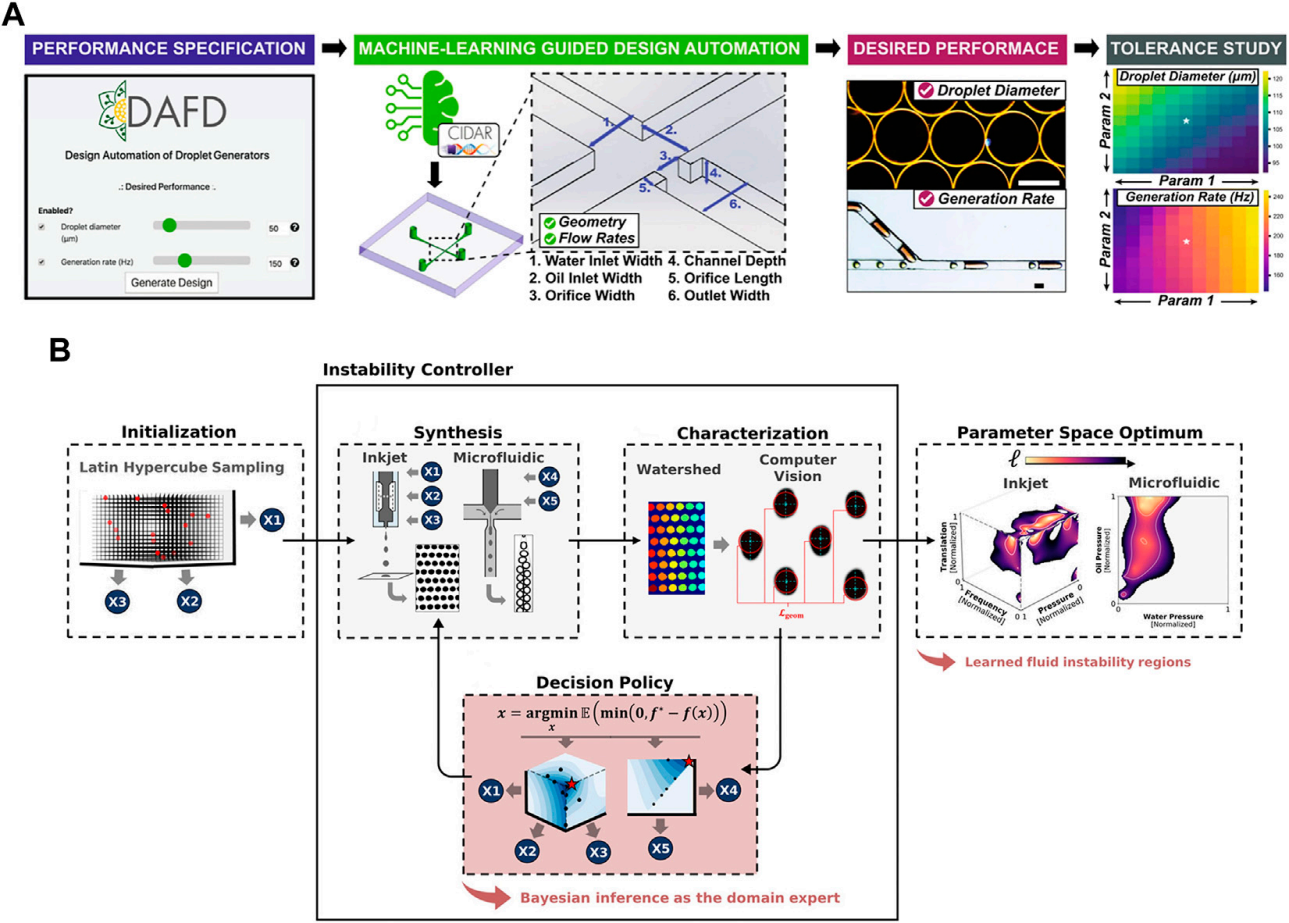
**(A)** The workflow of DAFD for flow-focusing droplet generators. The algorithms covert user-defined droplet diameter and generation rate into specific channel design and flow rates (Lashkaripour et al., 2021). Reproduced with permission from Springer Nature Publishing Copyright 2021. **(B)** Control feedback loop of the hardware–algorithm interface used for droplet optimization. The user captures an initial set of images of droplets generated by the device while varying experimental control parameters. The optimization software examines the data and generates a fresh set of control parameters for device usage, and this cycle is repeated iteratively (Siemenn et al., 2022). Reproduced with permission from ACS Publications Copyright 2022.

Furthermore, targeting at achieving design automation of fiow-focusing droplet generators, Lashkaripour et al. (2021) exploited machine learning to develop a tool named DAFD (Design Automation of Fluid Dynamics) (Figure 2A). A total of 43 droplet generators were analyzed to investigate the impact of various orthogonal dimensions and flow rates on droplet size and generation frequency. The generated dataset of 998 data points was utilized to train a neural network model, which could accurately predict channel designs based on user-defined performance criteria. This approach allowed for the estimation of droplet diameter and generation rate with errors within 10 μm and 20 Hz, respectively. Zhang et al. (2022a) utilized machine learning techniques and interpolation algorithms to design the inlet configuration capable of generating a customized concentration gradient of arbitrary nature. These methods provide assistance for further precise control over the concentration distribution. The introduction of machine learning has revolutionized droplet generator design, making it accessible to a broader audience and reducing the need for extensive expertise and design iterations in microfluidics. This development holds the potential to significantly reduce labor and experimentation costs. To achieve a desired droplet generation rate and size, Siemenn et al. (2022) combined Bayesian optimization with computer vision to automate the identification of stable droplet formation areas (Figure 2B). The deep learning loop effectively converged towards the user-defined performance using a total of 60 samples, and optimization procedure was completed within 2.3 h. This streamlined process significantly enhances the efficiency and precision in droplet behavior optimization. Raymond et al. (2020) used a DNN to design channel geometries capable of producing specific acoustic fields. This approach enables the precise manipulation and arrangement of microparticles and cells, allowing for targeted encapsulation and facilitating advanced studies in the field.

The “flow sculpting” technique involves using the pillar structure to shape the fluid into various geometric forms (Amini et al., 2013). Different arrangements of each pillar cause flow variations of the fluids, making the implementation of obtaining the structure sequence from microfluids a complex mapping relationship (Lee et al., 2019). Stoecklein et al. (2017) exploited the strong feature mapping ability of deep learning to establish a CNN. By inputting the fluid’s flow diagram into the network, the appropriate pillar type among 32 options can be automatically determined. In order to customize the concentration gradient more effectively, Hong et al. (2020) introduced a deep neural network (DNN)-based inverse design approach. This method aimed to establish a mapping between channel geometry and concentration gradient, where the simulated value of the concentration gradient was considered as the input, while the inlet pressure and sample concentration were chosen as the output variables. These works highlight the potential of intelligent data sampling to enhance the performance of deep learning models and suggest that similar approaches could be beneficial in tackling inverse problems in microfluidics.

In deep learning-aided droplet generation, fluid parameters (e.g., flow rate, viscosity, and surface tension), channel geometry parameters (e.g., width, height, and angle), and driving parameters (e.g., pressure and vibration frequency) collectively affect the predictions of droplet size (Hettiarachchi et al., 2021; Venkateshwarlu and Bharti, 2021). Training data limitations and model complexity can also impact the performance. When designing chip channel, important parameters include channel geometry, fluid characteristics, flow conditions (e.g., flow rate, pressure gradient, and inlet velocity distribution), and parameters related to focusing and mixing regions (e.g., blockage regions and mixer structures). These parameters determine the overall droplet generation performance (Huang et al., 2021; Prakash et al., 2022). It is important to note that while deep learning models provide valuable predictions and optimizations, their validation and optimization require expertise in physics, chemistry, and fluid dynamics. Additionally, high-quality and diverse data are crucial for accurate and generalizable models. Therefore, comprehensive data collection, rigorous training, and thorough validation are essential for reliable and effective results in both prediction and design tasks.

### 2.2 Droplet control

Different applications of microfluidics require unique processes, and microfluidic devices require design and optimization based on each study, which involves a variety of droplet manipulation and post-processing functions, thus advanced droplet control technology is needed for the conduct of correlational research.

To detect and track droplets in dense microfluidic emulsions, Durve et al. (2021) introduced an algorithm using deep learning techniques. The automated program integrated YOLO and DeepSORT deep learning models for droplet detection and tracking. The YOLOv5 model detected droplets in simulated images generated by Lattice Boltzmann simulations were employed to generate images for model training. The combined models demonstrate efficient detection and tracking of droplets even in the presence of significant deformations. Aiming at on-chip cell tracking and closed-loop feedback control for chip parameter modulation, Wang et al. (2021) developed an adaptive microfluidic system that integrated electrical sensors. Deep learning algorithms were employed to interpret real-time cell flow speed measurements from multiple locations. And a programmable pressure pump was also adjusted to maintain desired flow speeds. This system illustrate fast convergence even in the presence of external disturbances and has the potential to be used as a standardized biomedical test at the point of care, providing valuable information about the tested sample.

**FIGURE 3:**
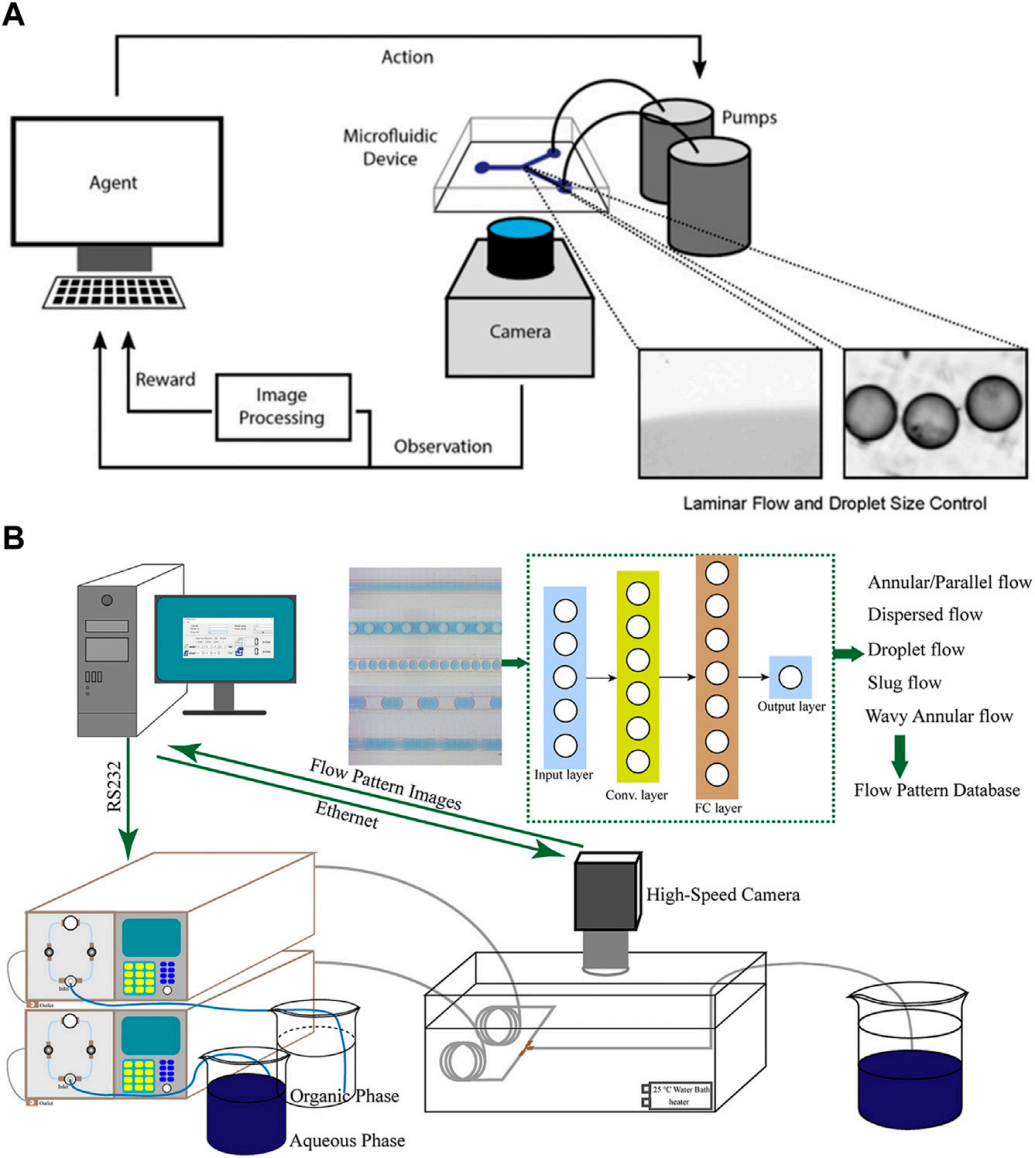
**(A)** The closed loop for autonomous flow control. The camera is employed to monitor the size of droplets, while reinforcement learning algorithms are developed to calculate errors and adjust the flow rates accordingly (Dressler et al., 2018). Reproduced with permission from ACS Publications Copyright 2018. **(B)** The experimental setup consists of an automated pump transport system, an online high-speed camera monitoring system, and a pattern recognition system based on CNNs. Two piston pumps were utilized to transport the organic and aqueous phases into the microchannel, which was connected to the outlet of a micromixer (Shen et al., 2020). Reproduced with permission from AIChE Copyright 2020.

Achieving laminar flow control and droplet size control are essential tasks in microfluidics, involving the precise positioning of the interface between miscible flows and the dynamic management of oil-infused droplet sizes in the segmented stream, respectively (Carreras and Wang, 2017). Corresponding to the two tasks, Dressler et al. (2018) employed a reinforcement algorithm based on deep Q network (DQN) and a model-free episodic controller (MFEC) to automate the control of volume flow in microfluidics (Figure 3A). These approaches realize automatic monitoring of flow rate and droplet size, and allow the detection of the error between the current and desired droplet size, thus enabling corresponding adjustment to the pumps to correct the flow rate. Shen et al. (2020) integrated a flow-pattern recognition model with online camera monitoring and automatic pump feeding systems (Figure 3B). The built CNN model enables the system to achieve real-time regulation of flow rates and generate desired patterns. Digital microfluidic biochips (DMFBs) possess the ability of manipulating discrete fluid droplets, but electrodes in the chips may degrade with the passage of time (Huang et al., 2020). Droplet transportation and operations associated with the degraded electrodes would fail, thereby affecting the integrity of the bioassay results. Liang et al. (2020) utilized deep reinforcement learning to achieve droplet routing on DMFBs. The devised model is able to detect electrode degradation and establish dependable routes for droplet operations in digital microfluidics. By avoiding droplets from contacting with degraded electrodes, the deep learning assisted droplet router has the potential to extend the lifespan of biochips, minimize the loss of valuable samples and reagents, and contribute to cost reduction in microfluidic experiments.

Simulating blood cells as elastic objects in the flow of blood plasma is a valuable approach for optimizing microfluidic devices for blood sample analysis. Bachratý et al. (2020) developed a neural network to predict the movement of red blood cells. The network learned from simulation data and provided comparable results to predictions based on fluid streamlines in simple box geometries. This system shows potential as a comparative tool for different modeled situations and can be valuable for analyzing videos of microfluidic flows in the future. In practice, achieving the expected Poisson distribution for encapsulation statistics can be challenging due to limited control over experimental variables and conditions. Gardner et al. (2022) employed YOLO (You Only Look Once) CNN architectures to develop an automated detector capable of identifying both whole droplets and individual cells within droplets. This automated detector enables the implementation of a process control feedback system to adjust experimental conditions effectively. Nevertheless, over prolonged periods, a notable decrease in the ratio of encapsulated cells was observed. This can be attributed to factors such as cell sedimentation or aggregation in the syringe.

In droplet control tasks involving flow regulation, fluid parameters, channel geometry parameters, and driving parameters remain crucial (Mehraji and Saadatmand, 2021). When assisted by droplet detection and tracking, image processing parameters like image size and resolution, as well as deep learning model parameters including network structure, layers, convolution kernel size, and activation functions, significantly influence detection and tracking accuracy (Li et al., 2021b). In droplet routing control, besides droplet motion parameters, routing rule parameters such as target position and path selection strategy are vital for achieving controlled droplet trajectories and velocities, thus ensuring stable and reliable microdroplet routing (Jiang et al., 2022). Therefore, when applying deep learning for droplet control, it is necessary to consider droplet driving mechanisms, chip channel characteristics, and make rational choices for model parameters. Adjustments and optimizations should be performed based on different experimental conditions to achieve precise and dependable droplet motion. Meanwhile, microfluidic droplet control typically requires rapid responsiveness and real-time performance. The training and inference of models in deep learning often take a considerable amount of time, which can lead to delays in real-time tasks. Additionally, deep learning requires a large amount of training data to train a model. However, in the field of microfluidic droplet control, obtaining a large-scale training dataset can be challenging due to the expensive equipment and intricate operations involved in microfluidic experiments. This limitation has resulted in many models being tested only in simpler simulated scenarios, and their capabilities still need to be validated in more complex non-simulated environments.

### 2.3 Droplet analysis

In some studies, it is necessary to analyze the reactions within the droplets, as well as the concentration of the droplet contents and the droplet status for more abundant information. The employment of deep learning makes the effective prediction of these features possible, which is conducive to improving the efficiency and performance of droplet based microfluidic experiments.

**FIGURE 4:**
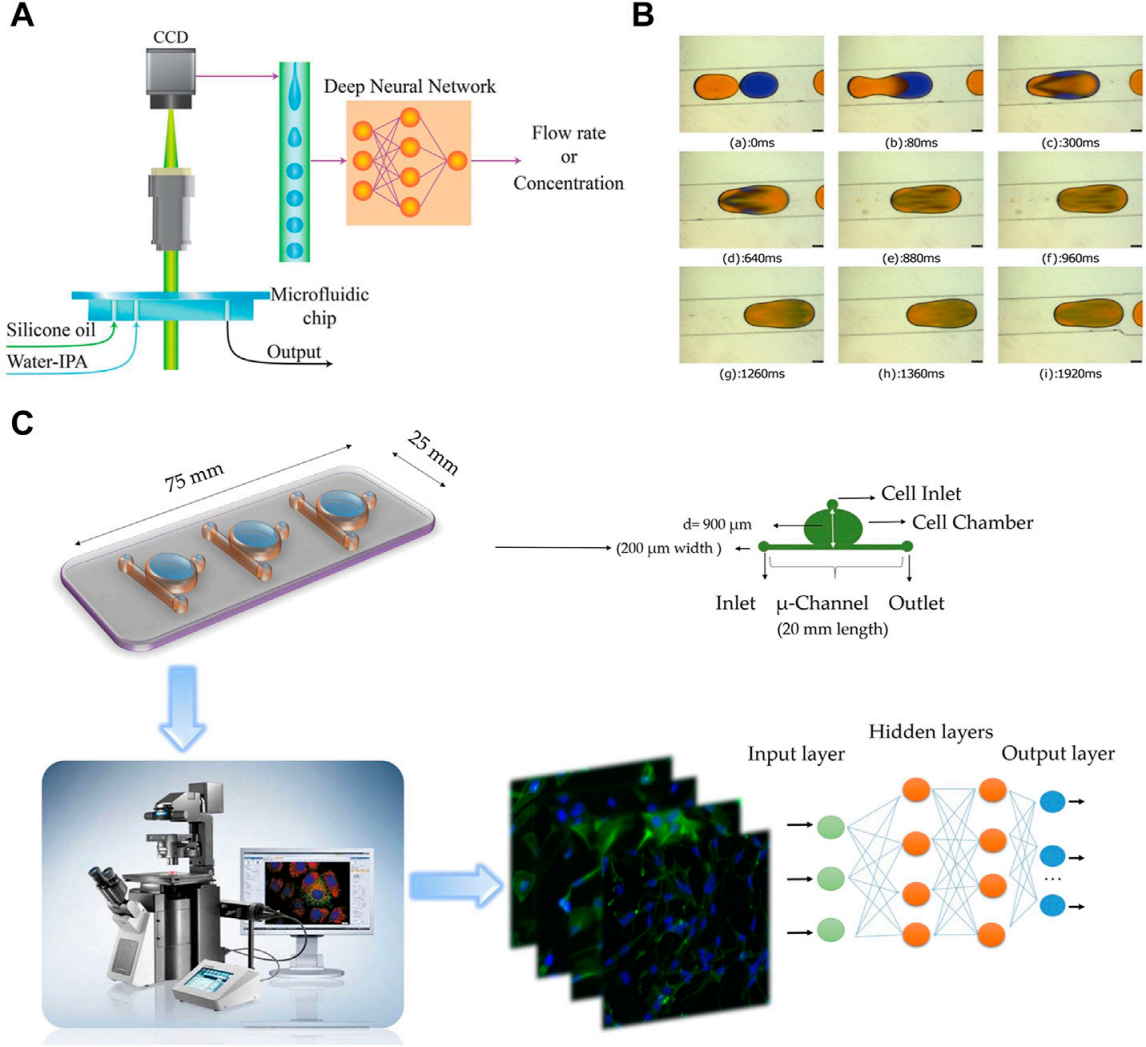
**(A)** The two-phase pattern of the droplet generation in a microfluidic device contains information about the fluid and the flow properties (Hadikhani et al., 2019). Reproduced with permission from Springer Nature Publishing Copyright 2019. **(B)** Sequence of images shows merging of droplets in a diverging channel using the “relative mixing index” method (Aijun et al., 2020). Reproduced with permission from AIP Publishing Copyright 2020. **(C)** Overview of the combined microfuidic deep learning approach consists of three main parts: a schematic representation of the microfluidic device employed for seeding lung cancer cell lines, cell imaging performed using IX-81 and IX-71 Olympus microscopes, and the classification of cell images into healthy cells or cancer cells using deep learning methodologies (Hashemzadeh et al., 2021). Reproduced with permission from Springer Nature Publishing Copyright 2021.

In order to monitor the mixing within droplets, Hadikhani et al. (2019) trained DNNs using a large dataset of images recorded under various conditions to monitor the mixing within droplets (Figure 4A). This approach enables accurate measurement of the concentration of each component and the flow rate of the mixture. In a similar work, Aijun et al. (2020) successfully detected and classified mixed droplets using trained deep neural networks (Figure 4B). The droplets were categorized into low mixing, intermediate mixing, and high mixing based on pattern recognition. A large dataset was created by generating binary droplets that could passively coalesce in specific microchannel geometries. The deep neural networks showed high precision in detecting and classifying the droplets, regardless of variations in fluid color, dye properties, and volume ratio.

To distinguish individual droplets in a microfluidic system, Bartunik et al. (2020) designed a low-cost and portable detector equipped with an infrared and a color sensor. The employed machine learning model realizes the distinction between different ink concentrations and characterization of droplets based on color and size. Zhang et al. (2022b) developed an advanced method for accurately measuring the size of microdroplets. The method utilized deep learning techniques for instance segmentation and boundary fitting, resulting in highly precise size distribution curves with a diameter measurement error as small as 0.75 µm. This approach also enabled the detection and measurement of overlapped droplets and small satellite droplets, which was not achievable with previous methods. From another point, Khor et al. (2019) created a convolutional autoencoder model to determine if droplets would break during injection. Using approximately 0.5 million images, they generated an 8-dimensional feature representation that describes the shape of droplets in a concentrated emulsion, achieving the prediction of droplet stability. Using a combination of a deep neural network (DNN)-based semantic segmentation model and circle Hough transform (CHT), Song et al. (2023) detected and quantified fluorescent droplets with a wide range of sizes. Accurate measurement was achieved even in cases of low fluorescence intensity and when the images were unfocused. This approach has potential applications in digital polymerase chain reaction (dPCR) analysis for absolute quantification of nucleic acid molecules.

**FIGURE 5:**
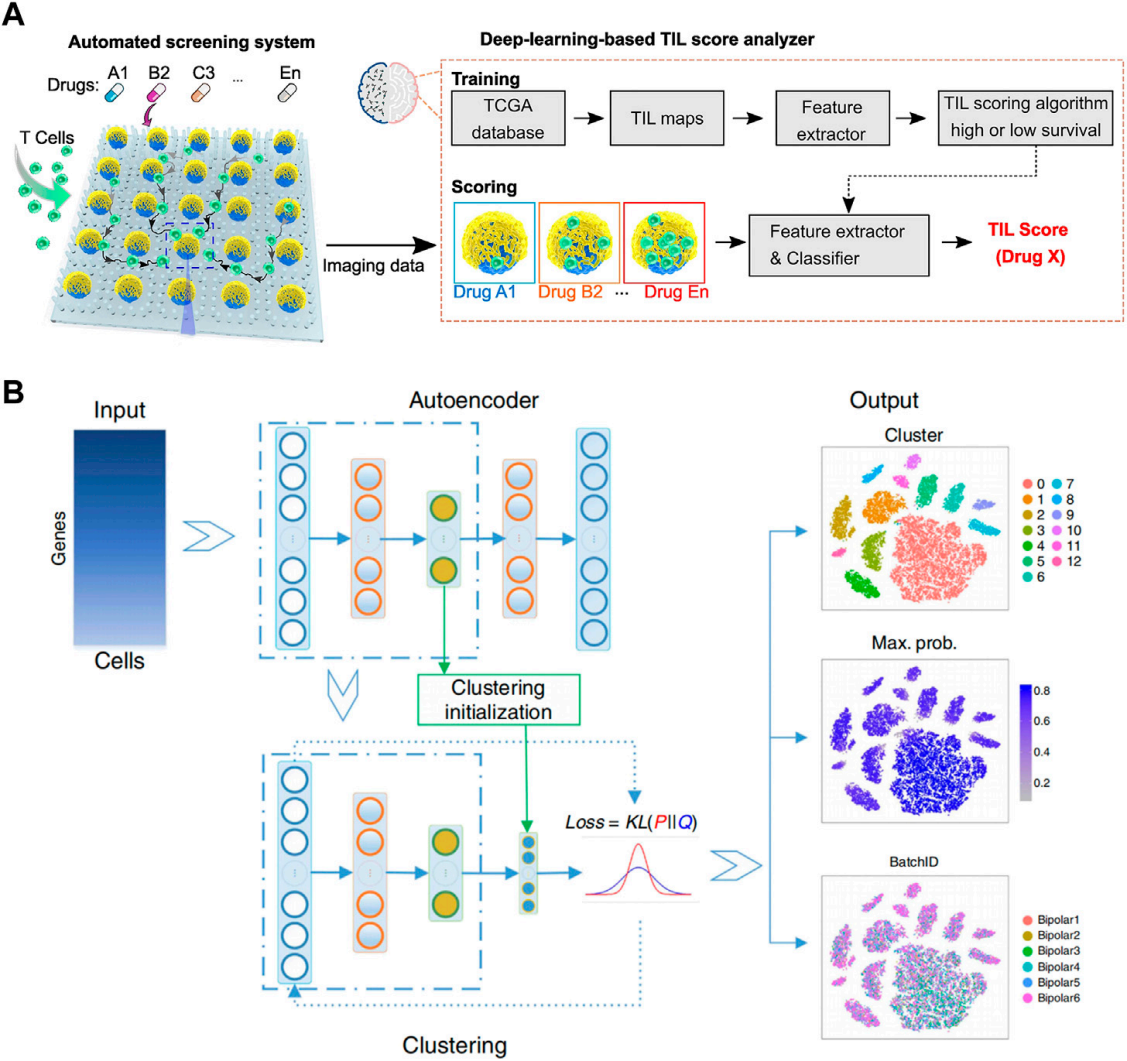
**(A)** Schematics of the automated screening platform for cancer immunotherapy screening, consisting of a deep learning TIL score analyzer that processes images and scores T cell infiltration patterns. These patterns are compared to TIL patterns observed in images from patient groups with high or low survival rates (Ao et al., 2022). Reproduced under Creative Commons license CC BY-NC-ND 4.0 https://creativecommons.org/licenses/by-nc-nd/4.0/. **(B)** Overview of the DESC framework. It begins by initializing parameters and using a stacked autoencoder to pretrain and create a condensed representation of the gene expression matrix. This encoder is then incorporated into the iterative clustering neural network to cluster cells in an iterative manner. The final output consists of cluster assignments, probabilities for each cell’s cluster assignment, and the low-dimensional representation of the data (Li et al., 2020). Reproduced with permission from Springer Nature Publishing Copyright 2020.

The combination of droplet analysis and deep learning is also extensively utilized in research pertaining to cells. Sesen and Whyte (2020) introduced a flexible and programmable microfluidic system for single-cell sorting. With supervised machine learning algorithms, droplets containing a single red blood cell can be differentiated from clusters by their distinct size and circularity characteristics. The system offers a valuable complementary approach for analyzing small cell populations or situations where labeling is undesired. Hashemzadeh et al. (2021) developed a computer-aided diagnosis system to distinguish between cancerous and healthy cells (Figure 4C). Lung cancer cell lines were grown in a microfluidic chip and stained for analysis. By utilizing deep learning algorithm, lung cancer cell line images were classified into different categories, achieving an impressive classification accuracy of 98.37%. Moreover, Ao et al. (2022) developed an automated microfluidic platform for high-throughput analysis (Figure 5A). By utilizing deep learning and clinical data, this platform allows simultaneous monitoring of T cell infiltration and cytotoxicity dynamics in 3D tumor cultures, and could assess treatment efficacy as well.

In order to improve the accuracy and efficiency of single cell sequencing, Lamanna et al. (2020) developed DISCO (Digital microfluidic Isolation of Single Cells for -Omics). The platform integrated digital microfluidics, laser cell lysis, and AI-driven image processing to capture individual cells from diverse populations. Genomic and transcriptomic analysis of the captured cells were carried out via next-generation sequencing. The platform provides a highly effective method for sequencing and can identify features at the single nucleotide variation level, comparable to state-of-the-art techniques. Also, using microdroplet technology, Fleming et al. (2019) employed a semi-supervised deep generation model for background removal in RNA sequencing to ensure the accuracy of counting. Li et al. (2020) introduced DESC, an unsupervised deep embedding algorithm that clustered scRNA-seq data by iteratively optimizing a clustering objective function (Figure 5B). DESC utilized iterative self-learning to gradually eliminate batch effects, as long as technical variations across batches were smaller than genuine biological variations.

In addition to image processing and model parameters, various droplet characteristics, such as size, shape, concentration, and data acquisition parameters, including droplet collection frequency and time intervals, are essential for effectively monitoring mixing and reactions inside droplets (Ghazimirsaeed et al., 2021). Different measurement algorithms can be applied based on the experimental scenario, such as pixel count-based calculations or physical property-based measurements. In droplet or encapsulated cell classification, a diverse dataset containing different droplet types and appropriate data preprocessing are crucial (LaBelle et al., 2021). Using data augmentation and optimized training strategies can also improve droplet classification performance. In single-cell sequencing, parameters for droplet image acquisition, such as resolution, exposure time, and focus, significantly impact subsequent processes (Petegrosso et al., 2020). Further, accurate droplet segmentation, extraction of single-cell images, and possibly dataset annotation are vital for precise sequencing of single cells within droplets. Overall, integrating deep learning into droplet analysis covers various aspects of intelligent microfluidic research. The role of different parameters varies across experimental scenarios, and extensive exploration is needed to achieve accurate, automated, and high-throughput analysis and experiments.

## 3 Problems and prospects

### 3.1 Raised problems in microfluidics integrated with deep learning

Microfluidics has the ability to intersect with different research fields, offering the opportunity to generate a wide variety of datasets for deep learning models. Conversely, deep learning can process the generated data to yield innovative and optimized solutions for microfluidics. Despite a wide range of promising applications of intelligent microfluidics, several under-developed and unsolved issues remain to be explored. Firstly, components in microfluidics can vary significantly from lab to lab, creating inconsistencies across the field that limit generalization. Deep learning model performance is only as good as the data the models are trained on, thus large batch variability limits the building of high-quality cross-institutional datasets. By training on a single lab’s data, the models are at a high risk of overfitting: building a dataset across a narrow distribution, models may perform well within the developer’s fabrication and operational workflow but poorly in others (Riordon et al., 2019). Secondly, deep learning techniques require representative data to build effective deep learning model for specific applications, therefore, acquired images need to be at satisfactory quality and quantity to build training datasets. Current research still heavily relies on high-precision and high-resolution image acquisition instruments in a laboratory environment. While it is possible to construct a more extensive and comprehensive dataset by making as many changes as possible to the environmental conditions under which the images were collected, this is not addressed in most studies (McIntyre et al., 2022). Thirdly, the high-performance capabilities often require high-quality deep learning models trained on massive amounts of data. As such, though deploying DNNs in high-performance and miniaturized hardware possesses additional benefits, it is still challenging (Srikanth et al., 2021). Lastly, extensive implementation of deep learning in microfluidics can require increased technical expertise for adopters. While tools with sophisticated GUIs are available, limitations in academic software maintenance can quickly render such tools obsolete before users are able to update the software for their own purposes (Pradhan et al., 2020).

### 3.2 The outlook for intelligent microfluidics

The proportion of scientists adopting machine intelligence into their laboratories will increase with the emergence of highly generalizable artificial neural networks that can be implemented without extensive retraining. Progress in cloud computing and the growth of computational power will also be significant contributors (Galan et al., 2020). Microfluidics leveraging machine-intelligence algorithms is thus expected to provide chemists with user-friendly platforms for high-throughput experimentation. The platforms can be implemented without demanding great expertise in deep learning to extract meaningful results (Dong et al., 2021). In the big data environment nowadays, data generated from low-cost pathogen-detecting paper microfiuidic devices by millions of globally distributed users could be paired with deep learning algorithms to track, predict, and ultimately contain disease outbreaks (Sun et al., 2023b). In addition to the detection of infectious disease and predicting rapidly evolving outbreaks, microfluidics may also play a role in a targeted distributed response. For example, microfiuidic systems could be applied to test and monitor food quality and safety throughout the food production chain, providing data-hungry deep learning strategies to contain and ultimately prevent contamination (Fu et al., 2021). Additionally, in supply chain, microfiuidics and deep learning are expected to be further combined with cloud-based distributed ledger systems, commonly known as blockchain (Guo et al., 2021). The combination would lay the foundation for building more powerful and intelligent blockchain applications, and is expected to have significant impacts in fields such as supply chain management and healthcare.

## 4 Conclusion

The application of deep learning in microfluidic systems has shown a strong trend of development, presenting significant advantages and practical effects in target detection, correlation prediction and result classification. With the rapid progress of big data, Internet of Things (IoT), blockchain, cloud computing and edge computing, artificial intelligence technology will gradually cover the microfluidic systems in data processing, status assessment, intelligent decision-making and automatic optimization. Despite the presence of some unexplored obstacles that require attention for continued advancement, the improvement of intelligent level in microfluidic systems is unstoppable. And intelligent microfluidics will find more extensive and significant applications in fields such as chemistry, biology, medicine, material science, and particularly provide assistance for the smart and high-precision analysis of biological samples.
